# Effect of Porcine Placental Extract Mixture on Alcohol-Induced Hepatotoxicity in Rats

**DOI:** 10.3390/cimb44050137

**Published:** 2022-05-01

**Authors:** Se-Mi Kim, Wen-Jing Diao, Wen An, Hyun-Jin Kim, Ha-Jong Lim, Keun-Nam Kim, Gun-Won Bae, Ju-Seop Kang

**Affiliations:** 1 Department of Pharmacology, College of Medicine, Hanyang University, Seoul 04763, Korea; huhuhu1015@naver.com (S.-M.K.); juice@hanyang.ac.kr (W.-J.D.); anwen@hanyang.ac.kr (W.A.); hope0211@hanyang.ac.kr (H.-J.K.); im147258@naver.com (H.-J.L.); 2 UBio, UNIMED Bldg #69, Samjeon-ro, Songpa-gu, Seoul 05567, Korea; knkim@unimed.co.kr (K.-N.K.); gwbae33@unimed.co.kr (G.-W.B.)

**Keywords:** alcoholic hepatotoxicity, pPEM (porcine placenta extract mixture), hepatic ADH, hepatic ALDH

## Abstract

This study was conducted to examine the effect of porcine placenta extract mixture (pPEM, enzymatic/acidic extract = 1/3) on alcoholic hepatotoxicity after pPEM dosing with alcohol in rats. The experimental groups were normal, control, silymarin, three pPEM (590, 1771, and 2511 mg/kg/day, po), and silymarin (100 mg/kg/day, po) groups (*n* = 10). Alcoholic hepatotoxicity was caused by a liquid ethanol diet for 4 weeks. The effect of pPEM and silymarin on alcoholic hepatotoxicity was evaluated by serology, hepatic ADH and ALDH activities, and histopathological findings. After oral dosing with alcohol for 4 weeks, ALT and AST were significantly increased to 33.7 → 115.6 and 81.37 → 235.0 in the alcohol group, respectively. These levels were decreased significantly to 83.9 and 126.7 in the silymarin group and dose-dependently to 73.6–56.9 and 139.2–122.8 in all pPEM groups. Hepatic ADH and ALDH might have been increased in the control and not in the silymarin and pPEM groups for hepatic ADH. All pPEM groups exhibited no effects on hepatic ALDH except for the high pPEM group. Mild inflammation and fatty lesions were observed in the alcohol group and were attenuated in the silymarin and pPEM groups. As a results, the pPEM showed protective activities against alcoholic hepatotoxicity on the serological markers, hepatic ADH and ALDH, and pathological findings.

## 1. Introduction

The placenta is the core organ of the fetus to develop and has several fundamental functions. After implantation, the fetus grows and develops in the mother’s womb. The placenta is full of essential nutrients for maturing the fetus and released outside the body after delivery, and it initiates maternal recognition of pregnancy, altering the local immune environment and maternal cardiovascular and metabolic functions through production of paracrine and endocrine hormones [1]. Human placenta extract (hPE) has been used to treat a number of liver diseases, including hepatitis and cirrhosis [2,3]. In related studies, hPE has immune-strengthening, antioxidant, and anti-inflammatory activities. It has been used as a treatment for various chronic pains, paralysis, osteoporosis, fatigue improvement, and even as a beauty treatment [4,5]. The main components of the placenta are essential amino acids, such as uracil, tyrosine, phenylalnine, L-tryptophan and collagen-derived peptides, vitamins, saccharides, nucleic acids, minerals, and hundreds of enzymes [6]. It is known to stimulate the functions of various cells and tissues of the body and to normalize various body functions by enhancing immunity-associated molecules such as interleukins (ILs) 1–4, which are essential for the body’s immune system, HGF (hepatocyte growth factor), FGF (fibroblast growth factor), and IGF (insulin-like growth factor) [7,8]. For more than 40 years, hPE has been prescribed clinically to treat chronic hepatitis, cirrhosis, and other hepatic diseases. In experimental animal models of hepatitis, hPE reportedly ameliorates hepatic injury, mediating liver regeneration and inhibiting inflammatory reactions and hepatocyte apoptosis [9,10]. High doses of placenta hydrolysates have been shown to ameliorate hepatic damage and induce regeneration in CCl4-induced liver injury in rats [9,11]. The hPE contains physiologically active substances showing antioxidant, anti-inflammatory, and analgesic effects. Due to its strong antioxidant substances, anti-inflammatory mediators, and growth factors, hPE may have a potential role in hepatocyte protection from several kinds of hepatic injury [12]. Placenta extracts are isolated from whole placenta using enzymatic hydrolysis or are hydrolyzed using placental villi alone [13]. The fermented porcine placenta has been developed as an alternative source to human placental extract due to biosafety concerns [14,15]. In particular, the porcine placenta is relatively safe and has been reported to have similar immune effects to human placentas [16].

Alcoholic liver disease (ALD) is a global human health problem [17] characterized by fatty liver, hepatitis, fibrosis, and cirrhosis. The frequency, dose, and duration of ethanol consumption are important in manifestation of the damaging effects of ethanol [18]. The healthy liver is resistant to the action of ethanol, and most individuals consuming alcohol have steatosis but not steatohepatitis [19]. In this regard, a binge-drinking habit in chronic alcoholics is one of the most important factors contributing to the progression of alcoholic liver injury [18,20]. In a recent study, binge alcohol consumption in rats was simulated by intraperitoneal administration of alcohol; dose- (1–5 g/kg body weight) and time- (1–4 h) dependent alterations in various parameters were monitored. Steatosis and necrosis (serum ALT) of the liver increased in 4 h, suggesting modest liver injury after acute alcohol treatment [21]. There is currently no satisfying treatment available for patients with ALD; dietary supplements have shown beneficial effects in animal models of ALD and might be useful in clinical practice. Dietary supplements such as animal placenta extracts have anti-oxidative and anti-inflammatory effects and therefore potentially alleviate liver injury in patients with ALD [22].

This study examined the effects of a single daily dose of porcine placental extract mixture (pPEM) on alcohol-induced hepatic damage in rats. To verify the efficacy of hangover extract of pPEM, the effects of ADH (alcohol dehydrogenase) and ALDH (aldehyde dehydrogenase) activities in the liver involved in alcohol metabolism, serum parameter profiles, and liver function enzymes were examined to determine the effects of hangover resolution and alcohol-induced hepatotoxicity in rats.

## 2. Materials and Methods

### 2.1. Materials

**Figure 1:**
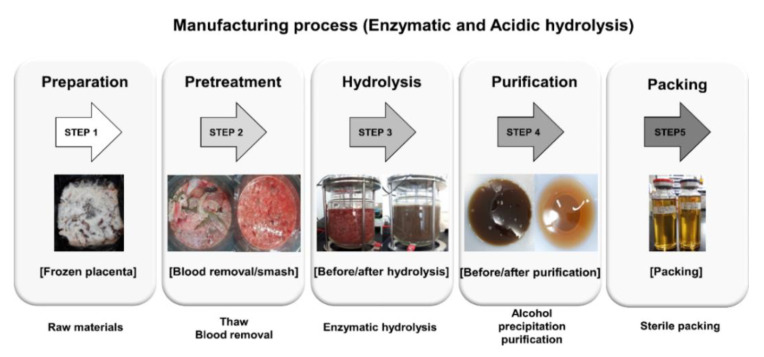
Raw material properties in the process steps.

The acidic and enzymatic pPEM used in this study was produced by UBio Co., Ltd. (Seoul, Korea). In brief, the weight of the pig placenta after removing aminons and cords was measured and chopped. The placenta hydrolyzate was prepared by dissolving the placenta materials, which had been separated and pulverized with a knife homogenizer (Daesung Arton, Korea) and Polyton homogenizer (ART-modeme Labortechnik, Müllheim-Hügelheim, Germany) by hydrolysis with protein-hydrolyzing enzymes (papain, etc.) or acid. The same amount of ethylacetate (*w*/*v* ratio 1:1) was mixed and extracted using a rotary wheel for 5 h. After centrifugation at 3000 rpm for 15 min, supernatants were separated through filtration and purification steps, sterilized in liquid (total nitrogen, 5.11 mg/mL), and combined with an excipient (maltodextrin) to form a pPEM powder (Figure 1). The dose of pPEM in this study was based on total nitrogen amount. The final products were characterized and are shown in Table 1. Among these formulations, we used the pPEM (*w*/*w* ratio of enzymatic extract/acidic extract = 1/3) (Table 1) to verify the effect on alcohol-induced hepatotoxicity in the rat.


(1) Amino acid (%)=(Amino acid Nitrogen MW)Amino acid MW× Amino acid ContentDilution Factor)×100

Peptide (%) = 100 (%) − mino acid (%)(2)

### 2.2. Experimental Groups

Eight-week-old male rats (Wistar rat, BW 250 g or less) were housed under a 12 h dark/light (Lux 200–300 Lux) cycle and assigned to groups of 10 rats each. Rats were allowed free access to a standard alcoholic liquid diet (Lieber DeCalie liquid ethanol diet). A liquid diet was prepared according to alcoholic liquid diet protocol [23]. Briefly, the diet was comprised of 860 mL water and 225.5 g ethanol combined with 132.28 g rodent powdered diet; a specific amount was supplied to each rat. Drinking water was not supplied separately according to the standard ethanol diet protocol. The health status of all animals was examined. After a 7-day quarantine period, weight change and general health status were observed, and healthy animals were used for the study. Animals were housed at a temperature of 23 ± 2 °C and a relative humidity of 50 ± 10% throughout the study. The experimental protocols were performed with approval from the Institutional Animal Care and Use Committee (IACUC) of Hanyang University (HY-2018-1-02). Free feeding of the liquid ethanol diet for 4 weeks induced alcohol hepatotoxicity. The rats were randomly divided into six groups. The normal group was fed a normal diet without any treatment. The alcohol group was fed a standard alcoholic diet (3 g/kg/day, *n* = 10) [24]. The silymarin group was treated with silymarin (100 mg/kg/day, po, *n* = 10) and an alcoholic diet [25]. The pPEM group (each *n* = 10) was divided into 3 groups treated with 590 mg/kg/day (L), 1771 mg/kg/day (M), and 2511 mg/kg/day (H) and an alcoholic diet, respectively. The pPEM was administered once a day at each dose during alcohol dosing to observe the therapeutic effect on alcohol hepatotoxicity.

### 2.3. Experimental Procedure

Animals were acclimatized for 1 week, and then the experiment was conducted for 4 weeks according to the protocol specified. The test substances were prepared orally once daily at doses of defined concentrations and amounts. The liquid ethanol diet was prepared according to the prescribed method of preparation and fed daily ad libitum. After 12 h of fasting in the fourth week, blood sampling for hepatotoxicity evaluation was performed by cardiac puncture and hepatic toxicity was evaluated by measuring hepatic enzyme levels after centrifugation at 10,000 rpm for 10 min. Portions of the tissues were collected to assess liver tissue pathology and hepatic ADH and ALDH activities.

### 2.4. Analytical Procedures

The general serum test was measured with a blood chemistry analyzer (IDEXX VetTest, Westbrook, ME, USA). Blood samples obtained from the tail vein were analyzed for alanine aminotransferase (ALT), aspartate amintrasferase (AST), alkaline phosphatase (ALP), serum bilirubin, lactate dehydrogenase (LDH), and total protein levels. ADH and ALDH activity colorimetric assay kits were used, respectively (Biovision Co., Waltham, MA, USA), to determine hepatic ADH and ALDH activities.

### 2.5. Histopathological Examination

Rat liver tissue was observed to be representative of each fragment in 3 of each group. Samples of rat liver tissue were subjected to general histological processes; tissues were fixed in 10% (*v*/*v*) neutral buffered formaldehyde, embedded in paraffin, and dehydrated with a graded ethanol series. Serial frontal 5 μm sections of the right lobe of the liver were stained with hematoxylin and eosin (H&E), followed by optical microscopy (Olympus BX53, Japan) to observe histopathological changes. Two pathological experts independently evaluated liver histopathology. The evaluation method was as follows: 0 if inflammation of the liver was not observed, 1 point if there were no more than 2 inflammatory lesions in the 200× field of view, 2 points if there were 2–4 inflammatory lesions in the 200× visual field, and 3 points if 4 or more inflammatory lesions in the 200× field of view were observed [26,27].

### 2.6. Statistical Analysis

Results are presented as the means ± SDs (*n* = 10). The statistical significance of analyzed multiple comparisons was determined using Duncan’s multiple range test with a significance level of *p* < 0.05.

## 3. Results

This section may be divided by subheadings. It should provide a concise and precise description of the experimental results, their interpretation, as well as the experimental conclusions that can be drawn. During the experimental period, body weight was measured once a week and the overall animal condition was examined in detail while measuring daily food intake. The pattern of weight change and food intake in each group was similar and there was no case of death due to an abnormal condition in each group.

### 3.1. Blood Chemistry Analysis

**Figure 2:**
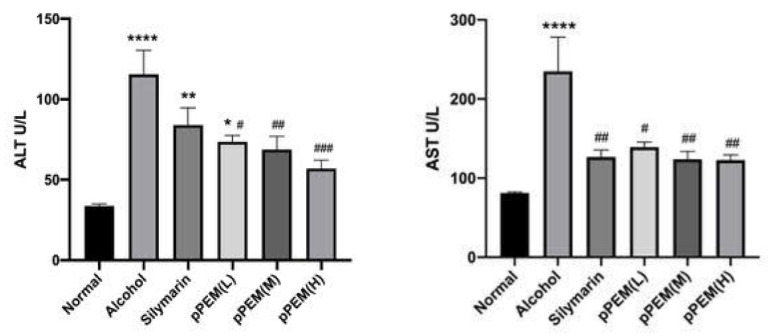
Effects of pPEM and silymarin on serum ALT and AST levels in alcohol-treated rats. The values are represented as means ± SDs (*n* = 10). The symbols in the figures show statistically significant differences among the groups as follows: * *p* < 0.05, ** *p* < 0.01, **** *p* < 0.001 vs. Normal Group; # *p* < 0.05, ## *p* < 0.01, ### *p* < 0.005 vs. Alcohol Group.

Serum levels of LDH (lactate dehydrogenase) are widely distributed in all tissues, especially in the heart, liver, and muscle. However, one cannot really expect a significant change in serum test values due to alcohol and pPEM administration. Here, we found that LDH levels were significantly higher in the alcohol group than in other groups. TG levels were increased significantly in the pPEM (M) and pPEM (H) groups. There was no significant difference between the groups for other markers (Table 2). The serum level of cytoplasmic enzymes such as ALT and AST is one of the important markers for hepatocyte damage. Serum ALT levels (IU/L) in the alcohol, silymarin, and pPEM (L) groups were significantly higher than in the normal group—by 343.3% after 4 weeks—and the values of all pPEM groups were significantly lower than the alcohol group—by 72.56% (*p* < 0.05). Serum AST levels (IU/L) in the alcohol group was significantly higher than the normal group—by 288.8%—and the values of the silymarin and all pPEM groups were significantly lower than the alcohol group—by 52.4% (Figure 2). The results indicated that cotreatment with silymarin and all doses of pPEM ameliorated alcohol-induced hepatic damage for 4 weeks in the rats.

### 3.2. Effects of pPEM on Hepatic ADH and ALDH Activities

**Figure 3:**
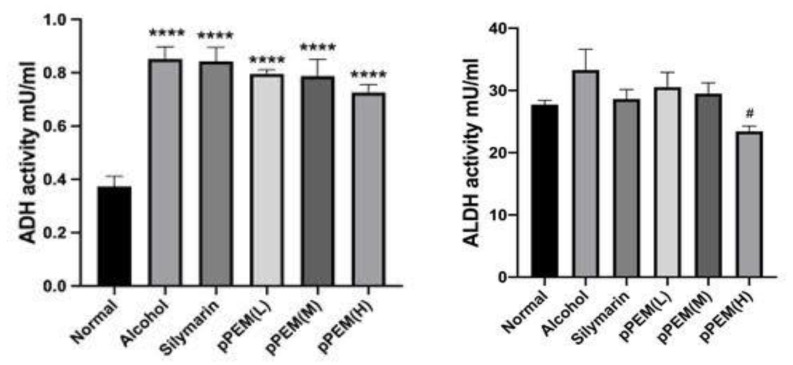
Effects of pPEM and silymarin on hepatic ADH and ALDH activities in alcohol-treated rats. The symbols in the figures show statistically significant differences among groups as follows: **** *p* < 0.001 vs. Normal Group; # *p* < 0.05 vs. Alcohol Group.

Hepatic ADH (mU/mL) activity was increased significantly from 139.05 ± 46.95 in the normal group to 340.54 ± 44.35 after alcohol dosing for 4 weeks. There was an overall increasing tendency in ADH activity in the silymarin and all pPEM groups (*p* < 0.05), indicating hepatic enzyme induction. The hepatic ALDH (mU/mL) activity was increased significantly from 28.09 ± 2.0 in the normal group to 57.01 ± 5.03 only in the pPEM (H) group (*p* < 0.05), indicating hepatic enzyme induction (Figure 3). The results indicated hepatic ADH enzyme induction caused by alcohol, silymarin, and pPEM, but ALDH in the pPEM (H) group was significantly lower than in the alcohol group.

### 3.3. Effect of pPEM on Hepatic Pathology

**Figure 4:**
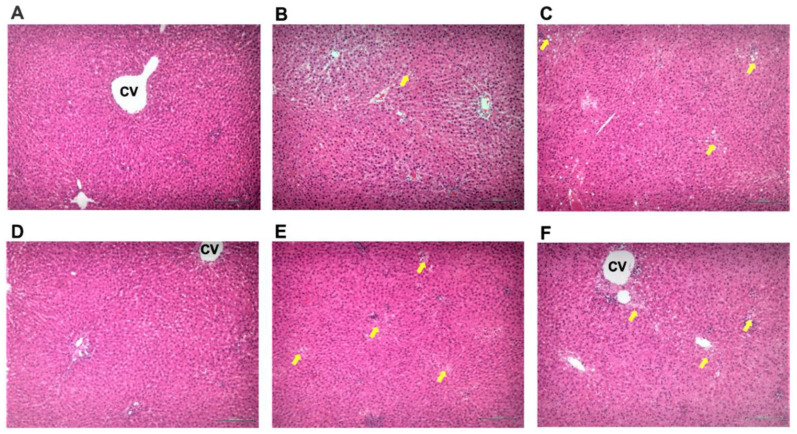
(**A**–**F**). Effect of pPEM treatment on alcohol-induced hepatic damage in rats using H&E staining of liver tissue: (**A**) normal; (**B**) alcohol; (**C**) silymarin; (**D**) pPEM (L); (**E**) pPEM (M); (**F**) pPEM (H). The experimental groups cotreated with alcohol and silymarin or a low dose of pPEM, a medium dose of pPEM, or a high dose of pPEM are labelled as pPEM (L), pPEM (M), and pPEM (H), respectively. CV, central vein. Yellow arrows indicate fatty degeneration.

Histological analysis could be used to reveal the extent of hepatocyte fatty degeneration, lobular inflammatory infiltration, and steatosis in liver. Figure 4 and Table 3 show representative images of liver H&E staining, histological analysis, and scoring of pathological changes in liver tissues in the different groups. Normal rats showed no pathological changes (Table 3, Figure 4A), but alcohol administration for 4 weeks caused fatty degeneration and mild inflammation and steatosis but no fibrosis in livers in the alcohol group (Table 3, Figure 4B). Otherwise, in the group administered with silymarin or pPEM, it was observed that liver lesions improved to some extent (Table 3, Figure 4C–F), that fatty degeneration was reduced to some degree, and that there was no inflammation or steatosis according to the pathological records system [11]. Histological examination of liver tissues further confirmed serum ALT and AST levels, indicating that pPEM had significant hepatoprotective effects in the alcohol-induced hepatic damage rat model.

## 4. Discussion

In this study, we showed that silymarin, which is a well-known hepatoprotective substance, and pPEM can be a potential protective agent for alcoholic hepatic damage in a model of alcohol-induced hepatotoxicity in rats.

The placenta is a rich source of many biological components, including hormones, cytokines, chemokines, and growth factors [28]. Many of these factors may act in an autocrine and/or paracrine fashion within the human placenta and regulate the production of other biologically active substances which may have potential as therapeutic agents, as suggested in previous studies, in which fractions of human placenta hydrolysate (hPH) stimulated tissue repair processes [29,30]. Over the last few decades, hPH has been prescribed clinically to treat chronic hepatitis [31,32], liver cirrhosis [12], and other hepatic diseases [33,34]. hPH reportedly ameliorates hepatic injury through liver regeneration [9] and inhibits inflammatory reactions and hepatocyte apoptosis [9,10]. Apoptosis and oxidative stress are crucial factors in the pathogenesis of acute liver failure and fulminant hepatic failure in severe stages of hepatitis [35]. hPH had a protective effect on hepatocyte apoptosis through antioxidative modulation and minimization of the autophagy process, which resulted in inhibition of D-galactosamine- and lipopolysaccharide (LPS)-induced hepatocyte apoptosis in in vitro and in vivo hepatotoxicity studies [36].

A high dose of hPH in hepatocytes ameliorated hepatic damage in carbon tetrachloride-induced liver injury in rats [37]. Hepatic ADH in the alcohol group was significantly increased, which may have been due to enzyme-induction by alcohol. Hepatic ALDH and gastric ADH were not changed, but gastric ALDH was significantly decreased only in the high-dose pPEM group [38]. A preclinical study with a concanavalin A-induced hepatotoxicity model demonstrated that hPE protected hepatocytes during chronic inflammation via suppression of intercellular adhesion molecule-1 (ICAM-I) and myeloperoxidase [10,39]. In addition, hPE increased superoxide dismutase (SOD) and decreased oxidative malondialdehyde (MDA) and nitric oxide (NO), which suggests that hPE has a protective effect on hepatocytes damaged by lipid peroxidation [10]. The protective effect of sheep placental extract (SPE) on concanavalin A-induced liver injury in balb/c mice confirmed that the protective effects against immune-induced hepatitis were largely responsible for the bioactivity of SPE [39].

In this study, serum AST and ALT levels (IU/L) in the alcohol group increased to values of 235.03 ± 113.98 and 115 ± 38.96 after 4 weeks, respectively, and were significantly higher than in the normal group. The AST and ALT values were reduced significantly in all pPEM groups and the silymarin group compared with the alcohol group. The hepatic ADH activity was significantly increased in the alcohol, silymarin, and pPEM groups (all doses) and this may have been due to enzyme-induction, but hepatic ALDH activity in the alcohol group was significantly higher than in the pPEM (H) group. The results indicated that pPEM has a potential protective effect against alcoholic liver damage by acting favorably on alcohol metabolism in the liver.

## 5. Conclusions

The silymarin and pPEM groups could suppress the alcohol-induced increase in ALT and AST levels, which are important serum markers of hepatocyte damage. Although inflammation, fatty degeneration, and mild steatosis were observed in the alcohol group, these pathological changes were suppressed in the silymarin and pPEM groups (all doses). The effect of pPEM and silymarin on hepatic ADH and ALDH activities showed that hepatic ADH activity was increased by alcohol administration due to hepatic ADH enzyme induction by alcohol. Silymarin and pPEM could induce ADH activity, like alcohol, to some extent. Hepatic ALDH activity was not affected by alcohol or silymarin, but it was significantly increased in the high-dose pPEM group and would have a favorable effect on blood acetaldehyde metabolism, which causes hangovers. From these results, the present study provides support for the beneficial effect of pPEM in suppressing and restoring hepatotoxicity by reducing the effects of alcohol on the liver or activating hepatic alcohol metabolism by enzyme induction.

## Figures and Tables

**Table 1:** Nitrogen, amino acid, and peptide amounts in the pPEM formulation.

Test Samples	Total Nitrogen *	Amino Acids	Peptides
Enzymatic Extract	46.8 mg/g	42.5%	57.5%
Acid Extract	35.8 mg/g	81.2%	18.8%
pPEM (E/A = 1/3)	39.0 mg/g	62.0%	38.0%

* For the ratio of amino acids to peptide contents, the total nitrogen and amino acid contents were analyzed and the peptide contents were calculated by the formula below for calculation for each content.

**Table 2:** Effects of pPEM on several serum biochemistry values in alcohol-treated rats.

Groups (*n* = 10)/ Parameters	LDH	TG	Albumin	ALP	Total Bilirubin	Cholesterol	Total Protein
	(IU/L)	(mg/dL)	(g/dL)	(IU/L)	(mg/dL)	(mg/dL)	(g/dL)
Normal	820.4 ± 127.98 ^a^	41.23 ± 28.94 ^a,b,c,d^	2.24 ± 0.13	483.6 ± 119.02 ^a^	0.14 ± 0.01 ^a,b^	0.14 ± 0.01 ^a^	5.32 ± 0.31
Alcohol	1162.64 ± 904.36 ^a,b^	32.97 ± 10.96 ^a,b^	2.38 ± 0.08	633.73 ± 91.97 ^b^	0.15 ± 0.01 ^a,b^	0.15 ± 0.01 ^b,c^	5.44 ± 0.14
Silymarin	646.7 ± 340.96 ^a^	26.2 ± 14.27 ^a^	2.37 ± 0.13	555.63 ± 108.75 ^a,b^	0.14 ± 0.02 ^a^	0.14 ± 0.02 ^c^	5.24 ± 0.28
pPEM (L)	695.97 ± 220.62 ^a^	39.44 ± 22.68 ^a,b,c^	2.33 ± 0.22	552.89 ± 94.56 ^a,b^	0.15 ± 0.02 ^a,b^	0.15 ± 0.02 ^a,b,c^	5.19 ± 0.46
pPEM (M)	818.59 ± 292.64 ^a^	68.21 ± 26.9 ^d,e^	2.34 ± 0.18	490.23 ± 82.2 ^a^	0.14 ± 0.01 ^a^	0.14 ± 0.01 ^c^	5.22 ± 0.34
pPEM (H)	783.81 ± 174.14 ^a^	64.56 ± 28.05 ^c,d,e^	2.39 ± 0.12	568.11 ± 85.03 ^a,b^	0.14 ± 0.02 ^a,b^	0.14 ± 0.02 ^a,b,c^	5.32 ± 0.27

Values are represented as means ± SDs (*n* = 10). Different superscript letters (^a–e^) are significantly different at *p* < 0.05 according to Duncan’s multiple range test. The experimental groups cotreated with alcohol and silymarin or a low dose of pPEM, a medium dose of pPEM, or a high dose of pPEM are labelled as pPEM (L), pPEM (M), and pPEM (H), respectively.

**Table 3:** Unweighted sum of scores for steatosis, lobular inflammation, and ballooning of hepatocytes in the different groups.

Lesions/Groups	Normal	Alcohol	Silymarin	pPEM (L)	pPEM (M)	pPEM (H)
Lobular inflammation	0	2	0	1	0	0
Fatty degeneration	0	2	1	1	1	1
Steatosis	0	1	0	0	0	0
Total score	0	5	1	2	1	1

## Data Availability

The data presented in this study are available in article.
